# Kin selection and polygyny: can relatedness lower the polygyny threshold?

**DOI:** 10.1098/rsos.140409

**Published:** 2015-06-09

**Authors:** Gaute Grønstøl, Donald Blomqvist, Angela Pauliny, Richard H. Wagner

**Affiliations:** 1 University Museum of Bergen, University of Bergen, Allégaten 41, 5007 Bergen, Norway; 2 Department of Biological and Environmental Sciences, University of Gothenburg, PO Box 463, 40530 Gothenburg, Sweden; 3 Konrad Lorenz Institute of Ethology, Department of Integrative Biology and Evolution, University of Veterinary Medicine Vienna, Savoyenstrasse 1a, 1160 Vienna, Austria

**Keywords:** kin selection, polygyny, relatedness, mate choice, lapwings

## Abstract

Resource polygyny incurs costs of having to share breeding resources for female breeders. When breeding with a relative, however, such costs may be lessened by indirect fitness benefits through kin selection, while benefits from mutualistic behaviour, such as communal defence, may increase. If so, females should be less resistant to sharing a territory with a related female than with a non-related one. We investigated whether kin selection may lower the threshold of breeding polygynously, predicting a closer relatedness between polygynous females breeding on the same territory than between females breeding on different territories. Northern lapwings, *Vanellus vanellus*, are suitable for testing this hypothesis as they are commonly polygynous, both sexes take part in nest defence, and the efficiency of nest defence increases with the number of defenders. Using an index of relatedness derived from DNA fingerprinting, we found that female lapwings that shared polygynous dyads were on average twice as closely related as were random females. Furthermore, relatedness did not correlate with distance between breeders, indicating that our findings cannot be explained by natal philopatry alone. Our results suggest that the polygyny threshold in lapwings may be lowered by inclusive fitness advantages of kin selection.

## 1. Introduction

Kin selection, or indirect fitness advantages arising from favouring relatives over unrelated individuals, is assumed to underpin a range of adaptive choices concerning whom to invest in and how much to invest [1–7]. In terms of fitness, an individual should benefit when a relative perpetuates their genes to further generations to an extent proportional to their relatedness [1]. It should therefore be more beneficial to contribute to the reproductive success of a relative than to an unrelated individual. Costs of interference and resource depletion imposed by antagonists should recede as their relatedness increases, reducing aggression and lowering the threshold for cooperation between individuals [1,7].

The amount of breeding resources offered by males determines on which territory a prospecting female should settle to maximize her fitness. Resource polygyny incurs costs for females in terms of loss of breeding resources to other female breeders on the territory. If a prospecting female finds that a territory, after subtraction of such costs, still yields more breeding resources than available bachelor-held territories, the polygyny threshold is exceeded and she should achieve greater fitness by breeding polygynously than monogamously [8–13]. The magnitude of the costs imposed by having to share resources is therefore a significant predictor of female settlement decisions in polygynous systems.

Females fight to avoid having to share breeding resources with other females (e.g. [14]), and finite breeding resources offered by males appear to be typical examples of economically defendable units subject to female competition [15]. The division of resources between females sharing a territory is likely to be dictated by relative differences in competitive strength. The stronger female may be able to control the larger share of the breeding resources on the territory, or prevent prospecting females from settling altogether [11,13,16–20]. The magnitude of the costs of sharing resources is thus likely to vary depending on the individual strength of the females competing for the breeding resources on a given territory.

Relatedness might prove to be an additional modifier of the sharing costs. Owing to inclusive fitness advantages, the expected costs of sharing a territory (i.e. the polygyny threshold) should be lower for related females than for unrelated ones. Further, any benefits of cooperation among polygynous breeders, like advantages of communal defence (e.g. [21]), would be boosted because contributions to the defence of offspring of related members of the dyad would increase the inclusive fitness of the breeders.

The northern lapwing (*Vanellus vanellus*, hereafter lapwing) is a socially polygynous shorebird, in which 20–50% of the males breed simultaneously with two to four females in the presence of unpaired males. They tend to aggregate in breeding clusters often consisting of 20–30 contiguous breeding territories [22–24]. Lapwings show bi-parental care, with males contributing to incubation, leading chicks to and competing for favourable chick foraging areas, as well as defence of eggs and chicks [24–30]. This species aggressively guards its offspring against predators and exhibits an efficient communal defence where the efficiency improves with number of contributing adults [25,28]. Polygynous males divide their parental effort between the broods, leaving the females to compensate for the loss in paternal care [24,27,29]. Resident lapwing females therefore often behave aggressively towards females prospecting for secondary status [27,31,32].

To examine the hypothesis that females share polygynous dyads with related females, thereby reducing the polygyny threshold, we compared genetic similarity (band-sharing from multi-locus DNA fingerprinting) between polygynous females that share a breeding territory with that between randomly matched females. No difference would indicate that no kin-selection effects were at work, whereas a higher relatedness between polygynous females sharing a territory compared with randomly matched females would support the presence of kin-selection effects. If, however, there is a correlation between proximity of breeders and genetic similarity, the above comparison might be confounded by natal philopatry. We thus also examined the correlation between inter-nest distances and genetic similarity of randomly matched females.

## 2. Material and methods

### 2.1. Field work

During 2003–2005, we studied lapwings on the island of Öland, Sweden, in several breeding clusters south of the village Stenåsa (56°31’07”*N*, 16°36′11′′*E*). The study sites were coastal pastures moderately grazed by cattle. We monitored 277 breeding territories, of which 117 (42.2%) were held by a polygynous male, 129 (46.6%) by a monogamous male and 31 (11.2%) of the territorial males were bachelors that did not succeed in attracting females (G. Grønstøl, D. Blomqvist, A. Pauliny and R. H. Wagner 2005, unpublished data). One of the polygynous territories (0.4%) was tetragynous, 17 territories (6.1%) were trigynous and 99 territories (35.7%) were bigynous. The number of breeding females per territorial male was 1.38 (or 1.55 when excluding bachelors).

During the study period, we trapped and ringed 120 breeding lapwing females on the nest. A small blood sample (20–50 μl) was collected from each individual by puncturing the brachial vein. The blood was suspended in Queen's lysis buffer [33] and stored at 4°C until subsequent DNA analyses.

Our sample consisted of 32 pairs of polygynous females breeding on the same territory (hereafter co-breeders), and 56 females that were randomly assigned to 28 pairs forming a control group (hereafter random pairs). In the random group, 33 of the females were monogamously mated, the mating status was unknown for one female and 22 females were polygynously mated. The latter were cases where we were only able to sample one of the breeding females in a polygynous dyad. We trapped females on their nest, so we were unable to trap both of them if one of their nests was lost due to predation before the trapping attempt. If predation rate correlates with relatedness between females breeding on the same territory, the estimate of relatedness in our sample of co-breeders may not be fully representative of polygynously breeding females in the population. Nest predation in our study population was almost exclusively due to nocturnal mammalian predators (fox, *Vulpes vulpes*, and badger, *Meles meles*), and we consider it very unlikely that the risk of predation is affected by relatedness between co-breeding females. Individual females were only represented once in the analyses.

Because co-breeding polygynous females generally bred in closer proximity to each other than the random pairs, we needed to know if relatedness (see below) correlated with the physical distance between nests to evaluate whether the comparison of co-breeders versus random pairs was confounded by relatedness effects of spatial distance. GPS coordinates were recorded for all nests, and these were used to calculate the distance between the paired breeders.

### 2.2. Genetic analyses

We estimated the degree of genetic similarity between females using band-sharing coefficients derived from multi-locus DNA fingerprinting. Band-sharing does not provide an exact measure of relatedness between individuals, but an index that reflects their relatedness. This has been repeatedly verified by studies comparing estimates of relatedness based on band-sharing with those derived from pedigrees [34,35]. Band-sharing can therefore be used for statistical testing of, for example, differences in relatedness between groups [34–38]. It should also be noted that indices of relatedness derived from different genetic markers, such as mini-(DNA fingerprinting) and microsatellites, are often correlated [37,39]. Furthermore, in a recent study band-sharing values were used for corroborating an increase in the population-level of relatedness, as indicated by field observations [40].

Our laboratory procedures followed previously published protocols (e.g. [37]). In brief, high molecular weight genomic DNA was isolated from whole blood using proteinase K and phenol/chloroform/isoamylalcohol extraction [41]. Co-breeders and random pairs were loaded in adjacent lanes on 0.8% agarose gels, together with a λ/*Hind*III size marker in the two outermost lanes. We size-separated 4–5 μ*g* of *Hae*III-digested DNA using constant-field gel electrophoresis at 1.2 V cm^−1^ for 40 h. DNA fragments were transferred to nylon membranes using Southern blotting [41] and hybridized with the multi-locus probe as in Shin *et al.* [42]. The probe was radioactively labelled with [^32^P] dCTP by random priming using the Prime-a-Gene labelling system (Promega).

Fingerprinting only allows for comparisons of genetic profiles in adjacent lanes on the same gel. Hence, the pairs of females to be compared were planned prior to running the gels. We could not rearrange individuals into new pairs after the laboratory work was completed.

### 2.3. Scoring of fingerprints and data processing

Fingerprints were scored by standard methods (e.g. [43]). Bands were scored conservatively, i.e. only clear bands were included and only unequivocal cases of band-sharing were scored as common bands. If there was any doubt that the bands were identical, the bands were scored as non-matching. Furthermore, we standardized the scoring by restricting it to the region between the wells and the size marker band denoting 4 kb (where individual bands were still clearly visible). The mean number of total bands scored for females in co-breeder pairs was 15.08 (s.d.=2.74, *n*=64), and the mean number of bands scored for females in the random pairs was 15.23 (s.d.=2.61, *n*=56). Band-sharing coefficients (*d*-values) were calculated as described by Wetton *et al*. [44].

Repetitions for the resampling test (following e.g. Andersson & Waldeck [5]) were made using the randomization function in Microsoft Excel 2003, and IBM SPSS Statistics 19 was used for other statistics calculations.

## 3. Results

**Figure 1.**
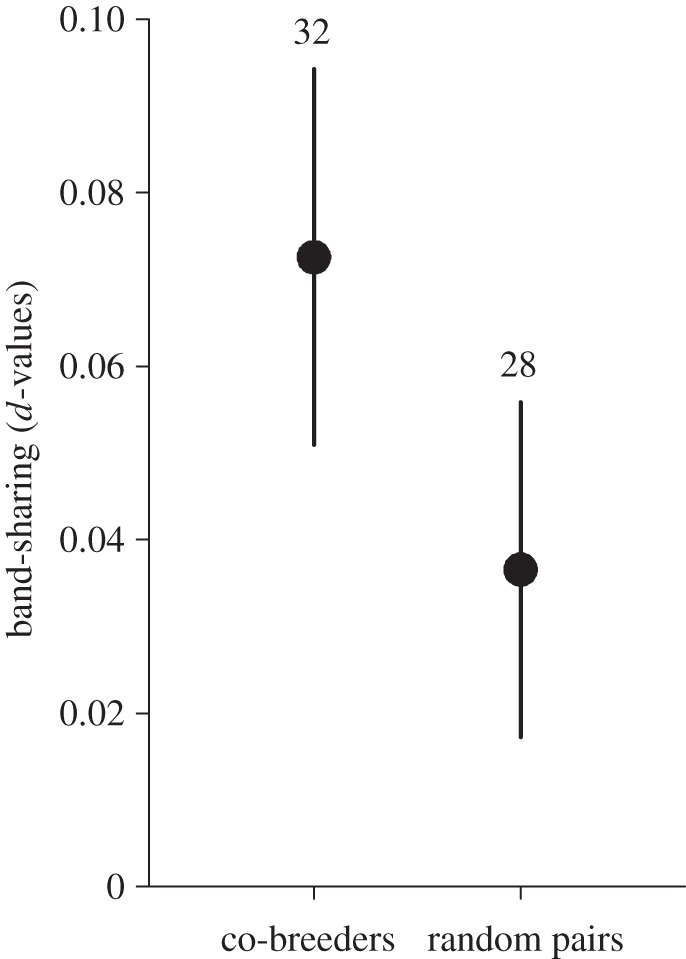
Relatedness for co-breeding females on polygynous territories and for females assigned to pairs randomly. Error bars indicate 95% confidence limits (*p*=0.015). Numbers above bars denote sample sizes.

**Figure 2.**
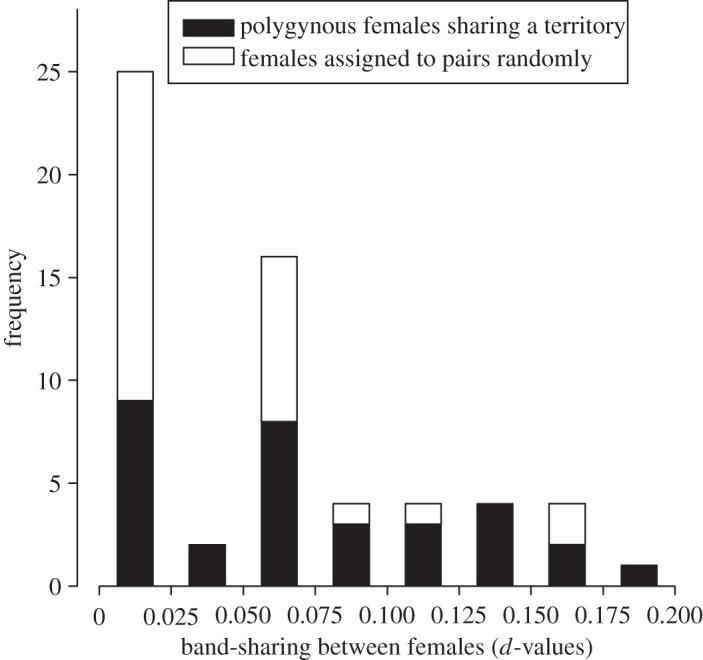
Frequency distribution of relatedness between co-breeding females on polygynous territories and for randomly matched females.

The average index of relatedness between polygynous females sharing a breeding territory was twice as high as in pairs consisting of randomly matched females (mean *d*-value for co-breeders: 0.073, s.d.=0.06, *n*=32; random pairs: 0.037, s.d.=0.05, *n*=28; figure 1). Furthermore, 21.9% of the co-breeder pairs had band-sharing values approximately corresponding to family relations in the order of first cousins to half sibs (greater than 0.125), while 28.1% did not share any bands. The equivalent values for random pairs were 7.1% and 57.1%, respectively. As the data were not normally distributed (around 42% of the values were tied at zero relatedness; figure 2), we statistically compared the two groups using a resampling test. We thus randomly resampled with replacement from the dataset 32 and 28 values, respectively, representing the sample sizes in the comparison. In 396 of 26880 random repetitions (the number of repetitions was chosen out of convenience), the absolute difference between the means of the two groups was larger than the observed difference in the original datasets of 0.036, yielding a probability of 0.015 that the observed difference had arisen by chance.

**Figure 3.**
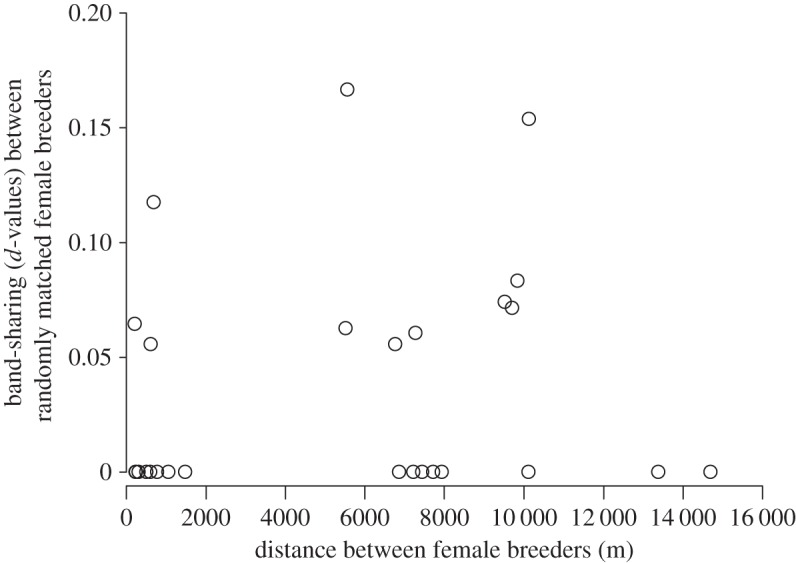
Relatedness between randomly matched female breeders did not correlate with distance between nests (Spearman's rank test: *p*=0.47, *n*=28 pairs).

If not opposed by choice mechanisms, natal philopatry could conceivably promote breeding with partners near the birth place, which over generations could produce a pattern where relatedness is correlated with spatial distance between breeders. To assess whether differences in proximity *per se* explained the observed pattern, we examined whether distance between random pairs of females was correlated with their relatedness, and found no evidence of this relationship (Spearman's rank test, *n*=28, *p*=0.29; figure 3).

## 4. Discussion

Polygynous females that shared a territory had on average double the band-sharing values of females that bred on separate territories. With random mating and strong natal philopatry, one might expect relatedness to correlate with proximity of breeders. Ringing studies have found that 61–72% of recoveries are found within 10 km from where they were ringed as chicks [45–47], so lapwings show a degree of natal philopatry. The higher index of relatedness between co-breeding females seems unlikely to stem from philopatry effects, however, because relatedness did not correlate with distance between nests. The results are thus consistent with the hypothesis that kin selection lowers the polygyny threshold in lapwings.

Our findings and conclusions about relatedness patterns are similar to results found in a study of conspecific brood parasitism in the common eider, *Somateria mollissima* [5]. In that study, the brood parasites and their hosts were more related than the average of the population, and relatedness did not correlate with distance between nests, which indicated that the genetic association was more likely to be structured by mating choices influenced by kin selection than arising from philopatry effects.

In a study of *Drosophila melanogaster*, Carazo *et al.* [7] recently found that relatedness between males competing over females in polyandrous associations inhibited male competition, resulting in increased male longevity, reduced female harm and increased female lifetime reproductive success, as predicted by kin-selection theory. These results indicate that the polyandry threshold in *D. melanogaster* is lowered by male relatedness, much in the same way as the polygyny threshold appears to be lowered by relatedness of co-breeding lapwing females.

Kin selection requires mechanisms to promote kin-association between polygynous females in a harem. Kin recognition has been found to occur in a range of birds and mammals [4]. Recent work demonstrates that young birds are able to discriminate between kin and non-kin based on olfactory cues alone [48]. Furthermore, lapwings are among the few bird species in which individuals differ in plumage characteristics to the extent that individuals can be reliably identified and monitored throughout the breeding season without the aid of individual colour ring combinations [22]. In fact, this study was prompted by our observation that some polygynous females resembled each other closely with respect to colour tones and speckling patterns in the face and on the breast. Such plumage traits could provide information on relatedness. As a capacity for kin-recognition is likely to enhance the precision with which females are able to choose the optimal breeding territory, such ability should be favoured by selection.

In breeding associations formed by kin, breeders may benefit from kin selection up to the point where it is balanced by costs related to inbreeding [49]. In lapwing dyads, inbreeding levels should remain low as long as the female breeders are only related to each other and not to their pair males. Lapwing males have a higher breeding site fidelity than females (38% higher return rates to the previous breeding site for males than for females; our unpublished data), and sex differences in philopatry and mating preferences may preclude close kin matings.

Studies have found evidence for nest parasitism and polyandry being favoured by kin selection [5,7]. Our lapwing study is apparently the first to link kin selection to polygyny. If the polygyny threshold is modified by kin selection, one might want to consider this effect alongside other candidate threshold modifiers when formulating predictions of optimal mate selection and reproductive output in polygynous systems. This may help to explain some of the mismatches between theory and empirical findings in tests of the Polygyny Threshold Model [11,13,14,50].

## Supplementary Material

Supplementary data for Kin selection and polygyny: can relatedness lower the polygyny threshold?

## Data accessibility

The dataset supporting this article has been uploaded as part of the electronic supplementary material.
